# Personalised Gait Recognition for People with Neurological Conditions

**DOI:** 10.3390/s22113980

**Published:** 2022-05-24

**Authors:** Leon Ingelse, Diogo Branco, Hristijan Gjoreski, Tiago Guerreiro, Raquel Bouça-Machado, Joaquim J. Ferreira, The CNS Physiotherapy Study Group

**Affiliations:** 1 LASIGE, Faculdade de Ciências, Universidade de Lisboa, 1749-016 Lisbon, Portugal; leoningelse@gmail.com (L.I.); djbranco@fc.ul.pt (D.B.); 2 Faculty of Electrical Engineering and Information Technologies, Ss. Cyril and Methodius University in Skopje, Skopje 1000, North Macedonia; hristijang@feit.ukim.edu.mk; 3 Instituto de Medicina Molecular João Lobo Antunes, 1649-028 Lisbon, Portugal; raquelbouca@gmail.com (R.B.-M.); jferreira@medicina.ulisboa.pt (J.J.F.); 4 CNS—Campus Neurológico, 2560-280 Torres Vedras, Portugal; 5 Laboratory of Clinical Pharmacology and Therapeutics, Faculdade de Medicina, Universidade de Lisboa, 1649-028 Lisbon, Portugal

**Keywords:** gait recognition, accelerometers, neurological conditions, motor impairments, personalisation, neural networks

## Abstract

There is growing interest in monitoring gait patterns in people with neurological conditions. The democratisation of wearable inertial sensors has enabled the study of gait in free living environments. One pivotal aspect of gait assessment in uncontrolled environments is the ability to accurately recognise gait instances. Previous work has focused on wavelet transform methods or general machine learning models to detect gait; the former assume a comparable gait pattern between people and the latter assume training datasets that represent a diverse population. In this paper, we argue that these approaches are unsuitable for people with severe motor impairments and their distinct gait patterns, and make the case for a lightweight personalised alternative. We propose an approach that builds on top of a general model, fine-tuning it with personalised data. A comparative proof-of-concept evaluation with general machine learning (NN and CNN) approaches and personalised counterparts showed that the latter improved the overall accuracy in 3.5% for the NN and 5.3% for the CNN. More importantly, participants that were ill-represented by the general model (the most extreme cases) had the recognition of gait instances improved by up to 16.9% for NN and 20.5% for CNN with the personalised approaches. It is common to say that people with neurological conditions, such as Parkinson’s disease, present very individual motor patterns, and that in a sense they are all outliers; we expect that our results will motivate researchers to explore alternative approaches that value personalisation rather than harvesting datasets that are may be able to represent these differences.

## 1. Introduction

People with neurological conditions (PNCs) often present abnormal gait patterns [1]. Concomitantly, gait has been shown to be a good predictor of PNCs [2].

A first challenge in gait analysis in free-living environments is that of automatically identifying gait (and non-gait) instances [3]. Recognising gait in free-living environments enables the calculation of macro and micro features—e.g., step variability and asymmetry [4,5]. The impact of classifying gait in free-living environments is severe to these endpoints, as the misclassification of a set of windows is likely to have a large effect on those micro characteristics and their interpretation [6].

The recognition of gait in free-living environments is commonly performed by assessing the movement data of accelerometers. Current methods can be split into two groups: wave transformation methods and artificial intelligence algorithms. Wave transformation methods [7,8,9,10,11] are based on recognising a general gait cycle. Artificial intelligence algorithms use previous data to train machine learning (ML) models [12,13], and lately, also Deep Learning (DL) models [14,15,16,17]. These models are then used to classify new data. Analysis aims include the recognition of activities, fall-prediction, and disease progression analysis.

Current wave transformation methods assume that every subject follows similar gait patterns. These expert knowledge systems assume a set of assumptions based on heel strike (HS) and peak detection [7,18]. Nevertheless, these algorithms tend to focus on capturing the patterns of a broader target population. However, fluctuations between and within subjects, a common aspect in PNCS, may not be captured when using a generic approach.

The current artificial intelligence paradigm, supported by the promise of *Big Data*, is focused on improving existing general algorithms, aiming to have a large enough dataset that is able to represent, and thus classify, anyone in the target group. General algorithms neglect the personal features of gait which are more strongly expressed for PNCs. However, gait is so personal that it is even used as a biometric to identify people [19,20]. The analysis results cover the complete dataset, ignoring individual results. We found, along with others (e.g., [21]), that the gait of many individual PNCs is not necessarily representable and therefore neither predictable nor analysable—by other PNCs’ movement data. Hence, a change of paradigm is presented herein, one that accommodates individual differences along with the challenges that harvesting large-scale datasets entail.

Training a personal algorithm for every person is not considered feasible, as these algorithms need a large, diverse dataset for training. Rodríguez-Martín et al. [22] solved this issue by building their personal detection model with both personal and general data but giving a bigger weight to the personal data. For every patient, a new model is trained, making it extremely time-consuming. To circumvent this inconveniently excessive time consumption, less-costly personalisation methods of general models have been developed. One method, as developed by Cola et al. [21], uses a second, more accurate, accelerometer that temporarily gives feedback to the model, which is being trained with data from the main accelerometer. This method requires an undesirably complex interaction between two devices.

Other promising work was conducted by Fu et al. [13]. To improve their models, they used personalisation with unclassified personal data. These data are first labelled using an improved pseudo-labelling algorithm, after which the models were enhanced for each participant with that personal data, which had a major impact on classification results. In the human activity recognition (HAR) of healthy individuals, transfer learning has been researched, showing promising results [23,24,25]. Mainly, these projects show that complex personalisation methods improve recognition for healthy individuals.

In this proof of concept, we show the relevance of personalisation for the gait recognition of PNCs. We train two of the most regularly used DL models for gait analysis, a Neural Network (NN) and a Convolutional Neural Network (CNN) [17]. The models classify accelerometer data into gait or non-gait data. This is a two-class problem, for the sake of simplicity. This could be extended to a higher number of movement classes [26,27] and tried with other classification approaches. The used data and the above described method and models are introduced in more detail in Section 2.

The general and personalised models were compared using a leave-one-out cross-validation method, similar to the method used by Bächlin et al. [28]. Promising results showing an improvement in overall accuracy: 3.5% for the NN; and 5.3% for the CNN. More importantly, we see the accuracy of individual participants with the most impaired recognition accuracy to largely benefit from personalisation; a maximum of 16.9% for NN; and a maximum of 20.5% for CNN; with only a few participants’ accuracies showing decreases (maximum of 2% for both models).

We compared the individual participant improvements with participants’ impairments. This showed that participants with high immobility had high personalisation improvements, showing the need for personalisation in for predictive models in order for them to be inclusive. In a population with neurological conditions (e.g., people with PD or stroke survivors), it is common for gait to be atypical. For a model to be representative of the individual differences in such populations, a general training dataset would need to be of large proportions, which is challenging (to say the least) to obtain in real environments. Furthermore, even such a model would likely end up falling short of capturing the whole population. Our results indicate that a general model fine-tuned with personalised data is able to increase recognition accuracy, making up for the differences each individual shows from the group. In addition to individual personalisation, these results also suggest that personalisation can be applied within the same person, as the disease fluctuates or progresses, enabling scenarios where personalised annotated data are collected at specific times (such as during a clinical appointment or in a semi-supervised way—e.g., prompts for activities with a mobile device).

## 2. Materials and Methods

The impact of the personalisation of DL models trained with the accelerometer data of PNCs was tested using a simple personalisation method. This personalisation method was applied to two DL models. These DL models were trained using data from 20 participants considered as PNCs. We start by introducing the data collection and data pre-processing. Thereafter, we put forward the two DL models. Lastly, we present the personalisation method.

As the paper is focused on the importance of personalisation in gait analysis of PNCs, we made some sub-optimal modelling decisions. We did not look for the best data format, features, model (type, size, and parameters), nor personalisation method, but used standard values from the state of the art. Research into the data format, feature selection, and modelling was conducted, and is still a hot topic of research. Improving the personalisation method for the gait analysis of PNCs is left to future research.

### 2.1. Data Collection

Study participants were recruited from the CNS—Campus Neurológico Senior, a tertiary specialised movement disorders centre in Portugal. Patients were eligible if they were diagnosed with a neurological disorder, had engaged in a specialised multidisciplinary program in CNS, and had agreed to participate. Each participant wore an Axivity AX3 accelerometer on their lower back during an hour of clinical assessments administered by a trained physiotherapist (Table 1) [29]. All sessions were video recorded for further analysis.

For this study, four standardised assessments of the participants were used to characterise the sample, namely the Movement Disorder Society’s Unified Parkinson’s Disease Rating Scale (MDS-UPDRS), the Hoehn and Yahr (H&Y) scale, the Schwab and England (S&E) Activities of Daily Living (ADL) scale, and the Mini-Best Test (MiniBEST). Further information on the assessments can be found in Table 2.

Our dataset included 12 patients with PD (9 males, 3 females); 2 stroke survivors (1 male, 1 female); 1 patient with epilepsy (female); 1 patient with polyneuropathy (male); 1 patient with Lewy body dementia (male); 1 patient with dementia (female); 1 patient with Alzheimer’s disease (female); and 1 person with mild cognitive impairment (male)—all aged between 56 and 90 years. More detail about the participants can be found in Table 3. Our data were collected during June and July of 2019. We mainly used S&E and MiniBEST to rate participants’ motor impairments, as these allow comparing participants across different pathologies.

Initially, we had 20 participants, but the accelerometer data showed problems due to malfunctioning of the sensor in three participants (P2, P15, P19). These participants were therefore excluded. Each second of the videos of the patients was manually labelled as gait and non-gait data. A total of 10 h, 14 min and 22 s were annotated. After alignment, a classified dataset was obtained. Notice that this annotation process is prone to errors. First of all, the annotation of the videos was performed per second, whereas gait does not necessarily cohere to partitions of full seconds. Second, the recognition of the start and end of gait slots is not straightforward and is vulnerable to subjectivity. We addressed these issues through the use of windows, explained in the following section.

#### Pre-Processing

AX3 accelerometer data were extracted with the open movement (OM) project *omconvert* [37]. Using the OM GUI, we were able to resample the data using linear interpolation and make the data at 100 Hz. Furthermore, we calibrated the data using the approach from Van Hees et al. [38] to guarantee that different devices have the same output under similar conditions. The obtained data contained acceleration data in three directions, representing the three dimensions of space, named *x*, *y*, and *z*, over time. We call each point in time an *instant*.

In some cases, the devices were not positioned in the same way. This resulted in the vertical axis being flipped. A simple multiplication of −1 with the vertical axis solved this issue.

After this, the data were split into windows, as is usual for activity recognition [39]. This split was done because gait cannot be detected from a single instant; it is a procession of multiple instants. We chose overlapping windows of 2 s and the distance between the starting points of windows was 0.4 s. Windows that were not unambiguously classifiable were removed from the dataset, leaving us only with windows that were either completely gait or completely non-gait. As such, transition windows were excluded, hence solving the subjectivity and per second video annotation issues put forward above. Excluding transitions, explicitly or by adding a moment’s “rest” between activities, is common practice (e.g., in Chong et al. [40] and Khan et al. [41]). Notice that transition windows are very interesting in their own right, but not especially interesting for this study.

Finally, before feeding the data into our models, we undersampled the skewed dataset. Basically, we randomly removed some windows which were classified as non-gait to obtain a balanced dataset. This is usual in ML techniques, as the prediction of less-represented classes is otherwise “underestimated” [42].

After the pre-processing steps, we ended up with window data from 17 participants. Each window contained 2 s of data, consisting of 200 instants, 10 ms apart, with acceleration data in 3 directions: *x*, *y*, and *z*. These windows were classified as either gait or non-gait. In total, we used 26,002 windows, among which half (13,001) were classified as gait.

### 2.2. Deep Learning Models

To test the hypothesis that algorithm personalisation is highly relevant for gait detection for PMIs, we trained two models: a simple (41 trainable parameters) Neural Network (NN) based on extracted features; and a more complex (2599 trainable parameters) Convolutional Neural Network (CNN). These models are commonly used for human activity recognition, where CNNs are probably the most commonly used model [17]. Other models that could be used, e.g., LSTMs, Random Forests, or Support Vector Machines, were excluded as they are beyond the scope of this paper which is focused on assessing the benefits of personalisation (as mentioned in Section 1). Additionally, studies have shown that CNNs have very good results compared to other models [43,44]. Both models predict whether a 2-second window of accelerometer data represents gait data or non-gait data.

#### 2.2.1. Neural Network Based on Extracted Features

The input of the NN is a set of 8 features extracted from each window. For each instant, the 3 directions, namely *x*, *y*, and *z*, are combined to form the vector magnitude (vm),

$$vm=\sqrt{x^{2}+y^{2}+z^{2}}.$$

The features extracted from each window are the mean and the variance of *x*, *y*, *z*, and vm, a total of 8 features. These features are commonly used, as studied by Chong et al. [40].

We built a simple NN with the input layer, one fully connected layer with 4 neurons, and an output layer. The activation function used was the sigmoid ($sigmoid\left(a\right)=\frac{1}{1+e^{-a}}$) for both non-input layers.

#### 2.2.2. Convolutional Neural Network

The input of the CNN included the complete windows. After the input layer, the first layer was a 1-dimensional convolutional layer, with 8 filters of size 32. This layer was followed by the conventional max pooling layer, for which we used a pool size and stride of 3. This was followed by a drop out layer with rate 0.2. The above layers were then repeated with the only change being the halving of the filter size of the convolutional layer; instead of 32 filters, we used 16 filters. After this, the data were run through a flattening layer. Lastly, we added a fully connected layer with 3 neurons, ending with the output layer. The convolutional, fully connected and output layers used the ReLU (relu(b)=max{0,b}) activation function.

In some cases, the CNN model became stuck in local error minima or saddle points. These models were discarded and retrained.

### 2.3. Personalisation Method

We used a form of domain adaptation to personalise a general algorithm. The general DL model was trained using the classified movement data of PNCs. The personalisation step consists of a second training session with PNC-specific data. This extra training was done with a small learning rate, so that the model was only tweaked. The general model is often referred to as the pre-trained model, whereas the personalised model is a fine-tuned version of the pre-trained model [45]. This method is called domain adaptation, which is a certain form of transfer learning. For our dataset, fine-tuning the pre-trained model (NN: 1 s, CNN: 3 s) takes considerably less time than training the general model (NN: 50 s, CNN: 285 s).

As mentioned previously, we have data from 17 participants. To test our personalisation method, we used the same leave-one-out cross-validation method employed by Bächlin et al. [28]. Practically, for each participant, we trained the pre-trained model with the data of the other 16 participants. After that, the model was fine-tuned using part of the personal data (data size averaged 925 windows). The other part of the personal data was used for validation. Validation was done both on the pre-trained model and on the personalised model.

## 3. Results

**Figure 1:**
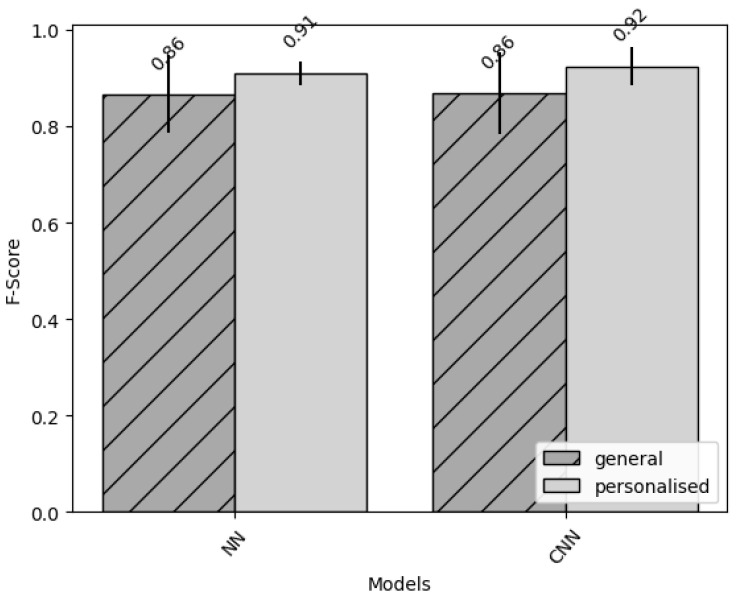
Comparison of the accuracy of general and personalised methods for both the NN and CNN models.

For every participant, we ran the personalisation method five times to average out inconsistencies. As we had a balanced dataset, we evaluated our DL algorithms with the accuracy metric, measuring the proportion of correctly classified windows [46]. For every participant, the average of the accuracy of the gait/non-gait classification for both the general and the personalised model were collected. These averages were compared, together with the overall accuracy average of all participants. On average, there was an improvement per participant for both models; 3.5% for the NN; and 5.3% for the CNN, as visualised in Figure 1. More interesting are the individual participants’ improvements. As mentioned previously, individual participants improved by a maximum of 16.9% for NN; and a maximum of 20.5% for CNN. Only a few participants’ accuracies showed decreases (maximum of 2% for both models). Compared to other studies, our personalisation approach has similar accuracy improvements over general models [21,47]. In Section 3.1 and Section 3.2, we look at the impact of personalisation on each individual participant for both models.

### 3.1. Neural Network Based on Features

**Figure 2:**
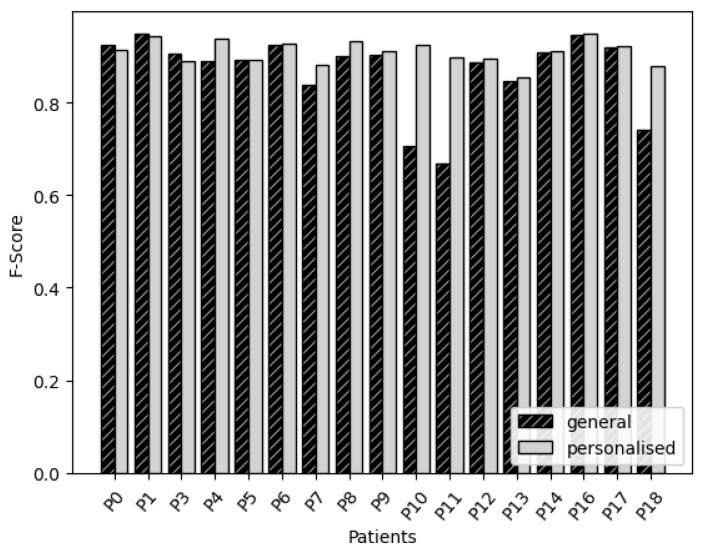
Neural networks: comparison of the accuracy of general and personalised models for each participant.

For the NN based on features, the accuracy of the general method and personalised method can be seen in Figure 2. Overall, we see that the personalised models perform better than or similar to the general model. The only exceptions are P0, P1 and P3, which have slight decreases in accuracy.

On the other hand, we see very large improvements for P10 and P11. Looking at Table 3, we notice that these are the participants with the lowest movement scores for most tests. For these participants, S&E score is very low, and the MiniBEST was aborted for P10, and scored as 0 for P11. P11 also had a high MDS-UPDRS score and the highest possible H&Y score. In summary, these are the participants that could be seen as the participants with the largest movement impairment. Other large improvements were observed for P4, P7 and P18. Both P4 as P7 have good scores overall, where P4 even has the best S&E score. P18 has a bad S&E score and the third highest MDS-UPDRS score, even though the MiniBEST and H&Y scores are average. In summary, the largest improvements are seen for the participants that are on the extremes of the spectrum of motor impairment, having either a large motor impairment or a slight motor impairment.

### 3.2. Convolutional Neural Network

**Figure 3:**
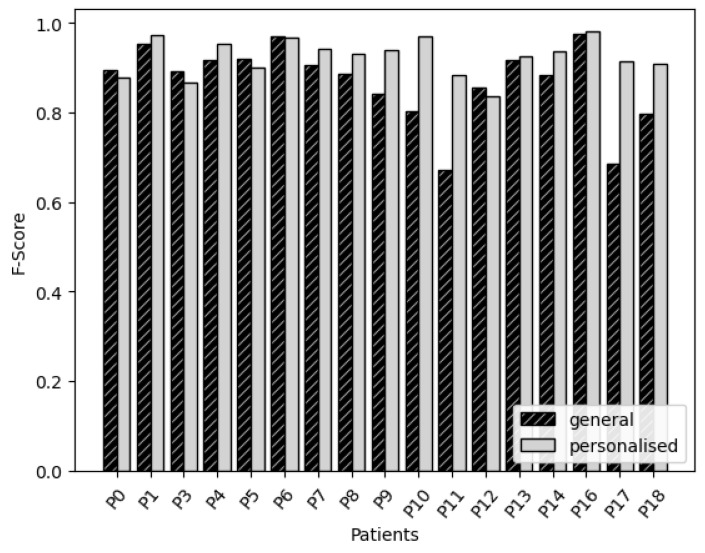
Convolutional neural networks: comparison of the accuracy of general and personalised models for each participant.

For the CNN based on features, the accuracy of the general method and personalised method were compared in Figure 3. Overall, we see that the personalised models perform better than or similar to the general model. The exceptions are P0, P1 and P6, which have a slight decrease in accuracy. There is a larger group of patients that significantly benefit from the personalisation method than for the NN based on features.

Large improvements were booked for P9, P10, P11, P17 and P18. As discussed above, P10 and P11 could be seen as the participants with the greatest movement impairment. Looking at Table 3 again, P9 and P18 have low S&E and MiniBEST scores. Furthermore, P18 scores quite high on the MDS-UPDRS score, but not so high on the H&Y score. P17 has average scores for S&E and MiniBEST but quite high scores for PD disease severity. Most importantly, we see that the personalisation step has the biggest effect on the participants whose gait was least accurately predicted by the general model.

## 4. Discussion

In the previous section, we showed the results of personalising two DL models, a simple NN and a more complex CNN. We found that the personalised models gave more balanced gait detection accuracy for all the different participants. The general models were worse at detecting the gait of participants with very low ADL and balance scores and high PD disease severity. We argued that these are the participants with the largest motor impairment. Furthermore, the general models had problems with detecting the gait of healthier participants. Overall, these participants can be seen as the outliers of our dataset. The personalised models drastically raised the model accuracies of these outliers, showing the importance of personalisation of DL models for the gait analysis of PNCs.

For DL models, one can argue that, given enough data and model parameters, one can train a model that predicts well for both general data and outliers. Apart from the obvious logistical problems (having enough data and training a large number of parameters), one should note the uniqueness of gait of PNCs. A general model is trained using general data that theoretically correctly represents future individuals. In the case of PNCs, all participants have a motor impairment and therefore are probably outliers. Hence, general data are unlikely to represent them.

Following the trend of *Big Data*, we are now witnessing the challenges of harvesting large datasets in challenging environments. Not only is collecting data from a diverse set of PNCs challenging in itself, but other issues arise including sharing and using these models among institutions. We argue for an approach that benefits from the advances of machine learning models but adapts to the scarcity of data by focusing on a personal one. The reason for this approach, variability between people, is also likely to apply for variations within the same person. For example, different sets of data can be used to personalise a model for different parts of the day (before and after medication), and personalisation can regularly happen to make sure that the model is evolving with the person’s condition.

In recent years, we have witnessed an increasing promise of objective outcomes in free-living environments with the goal of assessing and monitoring diseases, particularly neurological and neurodegenerative conditions. Gait has been one of if not the most relevant condition explored in this domain [48]. The approach that we present in this paper brings opportunities but also challenges. What is the future of personalised gait (and other activities) recognition systems and their application in real-life scenarios? We foresee scenarios, built on top of usable interactive systems, wherein patients are able to provide annotated data in controlled or uncontrolled scenarios and that enable the regular personalisation of models.

In this proof of concept, we showed the potential of personalisation in gait recognition for PNCs. The proposed method is simple yet effective. More complex methods could be more effective and should be explored. Methods such as those proposed by Cook et al. [23] and Ding et al. [24] should be considered. Furthermore, personalisation for clustered groups, a method proposed by An et al. [25], can easily be extended to our method. In their research, they cluster participants using *k*-means clustering, and they personalise for each cluster. This method eases the personalisation costs, but diminishes the performance improvement. We could do something similar, or use one of our standardised assessment measures to cluster. Furthermore, we could have different personalisation stages, where we first personalise per cluster, and then personalise per participant. This would be most beneficial for larger datasets.

These results and their implications should be of interest for researchers looking at gait in free-living environments as a relevant endpoint for neurological conditions. It calls the attention for personalisation approaches and launches opportunities for accurate machine learning approaches with *not-so-big data*.

## 5. Conclusions

Monitoring gait for PNCs is an emergent topic. Wearable inertial sensors open the opportunity to obtain more information about one’s gait in uncontrolled environments. To obtain valuable information from someone’s gait pattern, it is of utmost importance that gait instances are accurately recognised, even for people with abnormal gait. Current approaches are based on finding an expected gait cycle or training a gait classifier with data from others; these approaches are deemed to fail in the presence of unexpected gait patterns, a common occurrence in people with neurological conditions.

In our work, we compare general machine learning (CNN and NN) methods with a fine-tuned personalised version of each one of them. This approach enables a model to be trained with a not-so-large general model, and then personalised with individual data in a fine-tuning step. We showed that the latter improved the overall accuracy by 3.5% for the NN, and 5.3% for the CNN, and that those that were outliers (i.e., with the worst accuracy) in the results of the general version of the models were on par with the recognition accuracy expected from the larger group.

In this proof-of-concept, we encourage that personalisation be considered an avenue that can capture the different gait patterns and fluctuations in populations where differences are common and unexpected. This work opens opportunities for personalisation to each individual, but also for models to be fine-tuned for fluctuations from period to period (e.g., fine-tuning models to fluctuations that happen during the day).

## Figures and Tables

**Table 1:** Rundown of parameters used.

Type of Parameter	Measurement Tool
Demographic and clinical data	Clinical interview
Disease-specific symptoms	PD patients—MDS-UPDRS and Hoehn and Yard scale
	Stroke patients—STREAM and PASS
Disease severity	Patient global impression (PGI)
	Clinical global impression (CGI)
Gait	10 m walk test
Postural instability	Mini-Best Test
Functional mobility	The Timed Up and Go (TUG) test with and without a cognitive and manual dual-task
Physical capacity	2 min step test
	Five times sit to stand
Functionality in daily living	Schwab and England Activities of Daily Living scale
Kinematic gait analysis	Axivity sensors during the 10 m walk test (S&E)

**Table 2:** Rundown of standardised assessments.

Assessment	Evaluates	Pathology	Scoring
S&E [30,31]	Independence in ADL	Any	0–100: Higher S&E corresponds to a higher independence for ADL
MiniBEST [32]	Balance	Any ${}^{1}$	0–32: Higher rating in MiniBEST corresponds to a better balance
MDS-UPDRS [33]	PD disease severity	PD	0–200: Higher rating corresponds to higher disease severity
H&Y [34]	PD disease severity	PD	1–5: Higher rating corresponds to higher disease severity

^1^ Tsang et al. [35] researched MiniBEST on stroke survivors and Leddy et al. [36] researched MiniBEST on individuals with PD.

**Table 3:** Participants’ demographics and clinical information. G = gender; P = participant; YD = year of diagnosis; NA = not available.

P	Pathology	YD	S&E	MiniBEST	MDS-UPDRS	H&Y	Age	G
P0	PD	2007	80	29	39	2	56	M
P1	PD	2006	60	14	79	4	86	M
P2	Epilepsy	1954	Accelerometer problems				84	F
P3	PD	NA	80	29	49	2	79	M
P4	PD	NA	100	29	56	1	68	F
P5	PD	2004	70	24	93	2	75	M
P6	PD	2014	50	11	115	4	78	M
P7	Stroke	2019	70	30	-	-	65	M
P8	Polyneuropathy	2019	70	10	-	-	80	M
P9	Lewy body dementia	2011	40	14	-	-	79	M
P10	Alzheimer	2016	20	Aborted	-	-	81	F
P11	PD	2017	20	0	128	5	87	F
P12	Stroke	2018	80	25	-	-	78	F
P13	Dementia	2017	20	6	-	-	90	F
P14	Mild cognitive impairment	2019	40	20	-	-	89	M
P15	PD	2013	Accelerometer problems				70	M
P16	PD	2001	90	31	43	2	57	M
P17	PD	2008	60	24	90	4	67	M
P18	PD	NA	40	17	107	2	77	F
P19	PD	2009	Accelerometer problems				66	M

## Data Availability

The raw data supporting the conclusions of this article will be made available by the authors, without undue reservation, upon request.

## Abbreviations

The following abbreviations are used in this manuscript:

PNCs	People with Neurological Conditions
PD	Parkinson’s Disease
FOG	Freeze Of Gait
DL	Deep Learning
ML	Machine Learning
NN	Neural Network
CNN	Convolutional Neural Network
CNS	Campus Neurólogico Sénior
MDS-UPDRS	Movement Disorder Society’s Unified Parkinson’s Disease Rating Scale
H&Y	Hoehn and Yahr
S&E	Schwab and England
ADL	Activities of Daily Living
MiniBEST	Mini-Best Test
OM	Open Movement
vm	Vector Magnitude
